# The challenge of participatory projects for physical activity promotion: *What* to scale and *how* to scale?

**DOI:** 10.1371/journal.pone.0326737

**Published:** 2025-06-30

**Authors:** Raluca Sommer, Maike Till, Lisa Boyer, Philipp Weber, Alexandra Sauter, Susanne Ferschl, Anne Herrmann-Johns, Karim Abu-Omar, Klaus Pfeifer, Peter Gelius

**Affiliations:** 1 Department of Sport Science and Sport, Friedrich-Alexander-Universität Erlangen-Nürnberg, Erlangen, Germany; 2 Medical Sociology, Department of Epidemiology and Preventive Medicine, University of Regensburg, Regensburg, Germany; 3 BioPark Regensburg GmbH, Regensburg, Germany; 4 Department of Sport and Health Sciences, Technical University of Munich, Munich, Germany; 5 University of Lausanne, Institute of Sport Sciences/ Interdisciplinary Centre of Life Course Research, Lausanne, Switzerland; UnB: Universidade de Brasilia, BRAZIL

## Abstract

**Background:**

Scaling up participatory physical activity innovations is complex. Evidence on their nature and how they become embedded in health systems and policies is limited. This paper draws on four participatory research projects promoting physical activity among various populations and settings in Germany to explore their unique participatory innovations and strategies to scale-up.

**Methods:**

Document analysis and focus group interviews (n = 15) were used for a retrospective case study analysis based on the WHO-ExpandNet framework. 19 scientists of the project teams under study were approached and recruited by the authors during their collaborative work within the Capital4Health consortium. We performed a deductive content analysis to identify the innovation(s) scaled up, the implementers responsible for the process, and the context in which it took place.

**Results:**

The strategies employed differed depending on the intended scale-up direction: for the transfer to other sites (horizontal scale-up), projects usually scaled a participatory method; when working towards institutionalization at the systems level (vertical scale-up), they tended to scale specific actions for physical activity promotion, particularly those shown to be successful in the pilot phase of the projects. Overall, scale-up followed a strategic plan, but was continuously adjusted to setting-specific challenges and emerging opportunities.

**Discussion:**

Scaling participatory methods for physical activity promotion may require significant resources. Previously developed pilot actions seem to lend themselves to scale-up more easily, but may compromise the level of participation intended by the original project design. Within participatory research, successful scale-up will require deciding whether an innovation is appropriate for the intended context, whether it can address complex problems like health equity, and whether reducing participatory elements for the sake of improved scalability is acceptable.

**Conclusion:**

It is essential to develop novel theoretical concepts of scalability that account for the diversities identified within participatory projects observed in our study. The findings can guide future research on strategies to improve the effective implementation and scale-up or participatory physical activity innovations, ensuring that these remain adaptable and sustainable across various settings.

## 1. Introduction

While the number of effective interventions for promoting physical activity (PA) has significantly increased in recent years, only few have been widely transferred into practice to create broader impact [1]. Innovative and evidence-based interventions are often stuck at the demonstration project phase, i.e., there is no widespread implementation and thus no tangible public health impact at the sense of health-relevant behaviors change at the population level [2]. Given the alarming levels of insufficient PA worldwide [3] and within Germany with 74% children, 69% adolescents, 77% adults being inactive [4], this has drawn attention to the topic of scale-up. In this concern the German Federal Ministry of Health stated in 2013 that the true task is the sustainable adoption and transfer of innovative approaches [5].

Scale-up relates to how health innovations, proved successful in pilot projects, can be implemented on a larger scale to reach broader settings and population groups while becoming sustainably integrated into policies or systems [6]. A health innovation is a new or improved solution with the transformative ability to accelerate positive health impacts [7]. Within the context of PA promotion, an innovation can be a community intervention, a package of interventions, a certain practice, or even a specific intervention component that should be expanded to give more people access to PA [6].

Scaling up a health innovation requires intentional actions. For example, the Australian “Health Promoting Communities” program successfully *transferred* activities for healthy eating and exercise to multiple communities [8], a process known as horizontal scale-up [6]. Vertical scale-up, by contrast, occurs when the innovation is *institutionalized* by incorporating it into structures, policies, or systems, e.g., in the form of indoor smoking bans [9]. There exist examples of programs for PA promotion with large-scale rollouts at the state, regional, or even national level, but most of them have been developed within clinical, structured, or top-down (e.g., non-participatory) projects [10,11]. Evidence on scaled innovations emerging from participatory projects is scarce, and institutionalizing them is considered a challenge [12,13]. When community-based participatory research projects are successful and the interventions they develop to promote PA are effective, they tend to not only transfer the innovation they create but also the research methods underpinning the project. For example, the Our Voice Citizen Science Research Initiative is promoting their successful pilot-tested mobile health tool to involve citizens in the research process and, based on their input, to develop actions fostering a healthier community [14]. The mobile app and the research methodology used in the pilot phase are now being scaled worldwide within projects dealing with various health topics (i.e., mental health, PA, safety, violence prevention etc.). A similar trend has been observed in Germany, where projects using a specific participatory approach to promote PA, known as Cooperative Planning (CP; [15]) (see Section 2.1.), have transferred the methods employed along with other components of their projects to new settings.

In order to ascertain what can be scaled and how, it is therefore necessary to open the “black box” of participatory research for PA promotion to understand *(i)* how such methodologies influence the context in which they are applied, *(ii)* how participatory interventions are developed, and *(iii)* how both participatory methodologies and interventions catalyze further changes within the implementation setting. This will help to fill the gap in the existing literature on the nature of what is actually being scaled in participatory projects, the capacities of those performing the scale-up, and the specific environments to which innovations are being scaled [6].

In this concern, this paper aims to explore the unique participatory innovations and strategies for scaling up PA projects in Germany. We will first introduce the CP approach as an example for participatory methodologies to PA promotion, as well as the Capital4Health research consortium, which applied CP to develop PA promotion actions and subsequently initiate a scale-up to broader contexts. Building on a document analysis and focus group interviews, four case studies from Capital4Health will be presented to illustrate different planning and scaling strategies. The analysis is based on the WHO-ExpandNet framework [6] and focuses on *(i) what* was scaled to other contexts by the projects, as well as *(ii) how* participatory projects conducted the scale-up and which strategies they employed. The paper concludes by summarizing the potential of scaled-up participatory project innovations and available pathways to transfer and institutionalize components of such innovations for promoting PA.

## 2. Methods

In the recent decades, the “interactive knowledge-to-action” (IK2A) approach has gained increasing attention as it incorporates two central aspects of current discourses in health promotion research: a shift from deductive models of knowledge transfer towards interactive approaches and the trend towards co-production of knowledge and its translation into action. Drawing on various theoretical frameworks, such as knowledge-to-action [16], interactive knowledge translation, nexus theories [17], and transdisciplinary public health [18], the IK2A concept was approached through the implementation of CP, which fostered a progression from networking to joint collaboration and the development and implementation of shared agendas to promote physical activity through knowledge-to-action initiatives.

### 2.1 The cooperative planning approach

The CP is a research-driven participatory approach that engages various actors (e.g., researchers, policymakers, health promotion practitioners, population group representatives) in jointly designing, planning, and implementing tailored PA actions (or interventions) that fit to the preconditions of the setting and needs of the population group addressed [19]. The CP process typically consists of 6–8 meetings, which includes an initial assessment, brainstorming potential PA promotion actions, prioritizing ideas, and adopting an action plan for implementation. Action plans may include specific PA offers and sport activities (e.g., swimming classes), but also advocate changes to infrastructures or regulations that help promote PA.

In the German PA and health promotion field, the CP approach has been extensively used, and quite a few projects have succeeded in implementing such actions on a long-term basis [16,20]. The implementation experiences of projects gathered in the last two decades show how versatile the CP process – it changes the context in which it takes place, but also may get changed by the context, see CP’s methodological flexibility in the study of Abu-Omar and colleagues [21]. As such, one can see CP as a *method* to co-develop actions for promoting PA, such as sport offers, which are ultimately referred to as “PA interventions”. Due to its complexity [19], CP can also be considered an *intervention in its own right* [22], as the process has the potential “to transform the system due to its interaction with the context and the capability created from this interaction” [23]. However, the widespread use of CP across multiple projects, as either method or intervention, poses challenges for scaling up participatory projects using CP. It becomes difficult to determinate which elements belong to CP and are applicable to broader contexts, and which are context-dependent [23]. The question arises as to whether the CP approach itself represents an innovation that can be scaled up, along with its step-by-step implementation guidelines. Alternatively, we need to consider if the PA actions developed through the CP are the primary innovations that should be scaled up. In other words, is the focus on transferring that CP methodology, or should we prioritize scaling context-specific actions for promoting PA that emerge from this process?

### 2.2 The Capital4Health consortium

In response to the national objective of sustainably adopting and transferring innovative approaches, the German Federal Ministry of Education and Research provided political support by launching the Health Research Program, Action Plan for Prevention and Nutrition Research. This initiative offered funding for three years, with the possibility of an additional three years, to support research consortia focused on primary prevention and health promotion [24].

Capital4Health (C4H) is one of the research consortium that received funding in two phases: from 2015 to 2018 (Pilot project phase) and 2018–2023 (Scale-up phase), with a total of 2,8 million Euros as part of an effort to strengthen national research on prevention. Its four empirical projects – A4M (“Action for men”), health.eduPLUS (“Health-related Education”), PArC-AVE (“PA-related Health Competence in Apprenticeship and Vocational Education”), and QueB (“Qualität entwickeln mit und durch Bewegung/Developing Quality with and through PA“) – focused on promoting PA. As shown in Table 1, the projects varied in setting, population groups, goals, and outcomes.

**Table 1:** Overview of projects, intervention settings, sites, target groups, and pilot phase-developed actions and outcomes.

	A4M	health.eduPLUS		PArC-AVE		QueB
Setting	Community	University	School	Nursing school	Car manufacturer	Childcare center
No. site(s):						
*Pilot project phase*	*n* = 2	*n* = 2	*n* = 4	*n* = 2	*n* = 1	*n* = 12
*Scale-up phase*	*n* = 1 *(new community within new region)*	–	*n* = 4 *(new school form, i.e., primary school)*	*n* = 2 *(new school form, i.e., state nursing school)*	*n* = 1 *(new division of the car manufacturer, i.e., production)*	*n* = 18 *(new childcare centers within two regions)*
Groups addressed:						
*Target group*	Men over 50 years of age	Pupils	Pupils	Trainees	Trainees	Children
*Professional group*	Local stakeholders	Teachers, Lecturers	School board PE teachers in training	Trainers VE Teachers	Staff	Staff
Aims	Improve opportunities for PA	Increase teachers’ competences for physical education (PE)	Increase sport-related health competence of pupils	Increased Physical Activity-related Health Competence (PArC)		Increase PA levels
Actions for PA promotion developed in the pilot phase (Source)	Communication campaign (incl. fly ers) Low-budget PA program (i.e., SportCard) [25–27]	Competence-oriented lessons Teaching content	Annual fitness day for 9^th^ grade School-intern sports curriculum Methodologic-didactic actions [26,28]	PA class (BuG course); Continous education course for the VE Teachers	PA classes PA trainer workshops Tutoring system [29]	Kita Check-App/Quick-Assessment-Tool [30]
Outcomes of the pilot phase (Source)	Increased organizational capacity [25,26]	Improved PA-related health literacy and sport-related health competencies of students		Increased organizational capacity		Improved skills of nursing school teachers Higher PA levels of children and staff [30,31]

*VE vocational education; PArC physical activity-related health competence; PE physical education; PA physical activity; BuG “Bewegung und Gesundheit/Physical activity and health”*.

In the pilot phase (2015–2018), the four projects used the participatory CP approach to co- design and co-develop tailored PA-promoting actions, such as media campaigns, infrastructure improvements, group-specific PA, health programs or courses, and capacity building among key stakeholders (i.e., teachers) [19,32,33]. The participatory nature of the projects allowed various groups (e.g., target group, stakeholders, researchers, professionals) to work together, developing various for PA promotion that reflected their unique resources, expectations and visions for enhancing local PA promotion within their settings. Given the effectiveness of the CP approach in fostering engagement and generating impactful actions, it was further recommended for use in the second phase of the consortium.

In the scale-up phase (2018–2022), the projects aimed to scale the CP approach and/or components of their pilot projects to broader population groups and further settings. As shown in Table 1, the types of setting remained broadly the same (i.e., community, school, childcare center, car mechatronics), but varied in their specific forms (e.g., transitioning from elementary to primary school, or from private to state nursing schools). These differences introduced new underlying conditions for scaling up within the new sites compared to those in the pilot phase. Given the varied setting and their distinct conditions and capacities for promoting PA, the projects prioritized designing and planning the process in close collaboration with the local policymakers, professionals, and population groups of each new implementation setting, rather than simply “transferring” the innovations form the pilot phase. This approach aimed to ensure that both the scale-up process and the PA promotion strategies would be well-suited to the unique contextual conditions of each new site.

### 2.2 Research design

We used a qualitative research design to investigate the key elements of scale-up, particularly the innovations (see below) scaled within the empirical projects and strategies for transfer and institutionalization. For this, we relied on different types of data collected from various perspectives and at various points in time, thus ensuring a higher quality of results [34–41] The work by Tong and colleagues [35] guided our reporting of qualitative findings.

**Fig 1:**
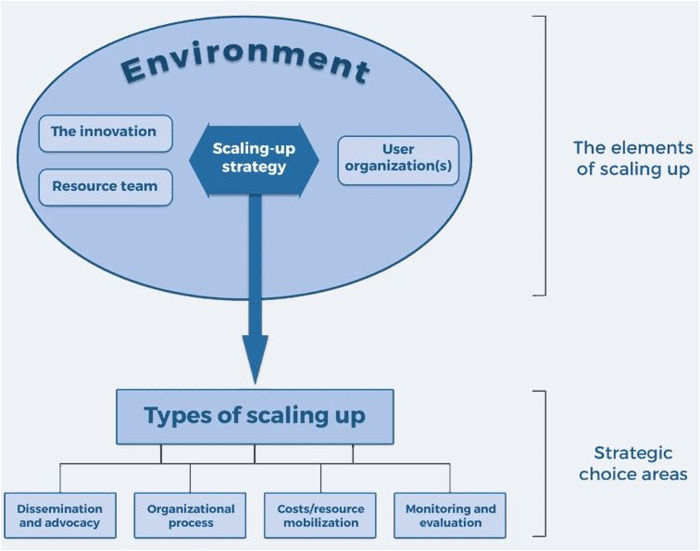
The WHO-ExpandNet framework for scaling up (6, p.5).

Based on our previous research on scale-up in PA promotion [36], we identified the WHO-ExpandNet framework [6], initially designed to help with planning scale-up, as a suitable framework to retrospectively analyze such processes [37,38]. The WHO-ExpandNet framework (see Fig 1) enables the identification of the following key elements: (a) the *innovation* (“What was actually transferred to other contexts?”); (b) the *user organization(s)* responsible for implementing the innovation on a larger scale (“Which entities adopted the innovation and how did this influence its scale-up?”); (c) the *resource team* that tested the innovation and sought to promote its wider use (“Which entities, familiar with the innovation from its pilot testing, supported its scale-up?”); (d) the *context* in which scale-up took place (“What was the socio-cultural-political and economic context in which scale-up occurred, i.e., roles, interactions, and relationships?”); and (e) the different *strategies* adopted for scale-up.

### 2.3 Data collection

We based our research on the consortium’s documentation (in particular, the grant proposal of the scale-up phase phase) and semi-structured focus group interviews (n = 15) with the researchers of the four projects scaling up the innovation. In total, 19 scientists participated in the study (n = 4 from A4M; n = 6 from health.eduPLUS; n = 4 from PArC-AVE; n = 5 from QueB). The authors of this study were also researchers within the Capital4Health consortium, who informed participants about study’s objectives and recruited them during collaborative work within the consortium.

The grant proposal included both a general description of the consortium’s focus as well as specific objectives and workplans of each project. Its analysis allowed for a systematic review of the projects’ intended scale-up targets and activities. One researcher (SF) conducted two rounds of interviews (n = 10) with project teams on different topics (e.g., project status, project’s collaboration partners, and key challenges associated with different research tasks). Participants were recruited from March 12^th^ until March 26^th^, 2019, and the first round of face-to-face interviews (n = 5) lasted ∼60–85 min. For the second round of interviews (n = 5), participants were recruited from January 15^th^ until March 18^th^, 2020. Due to the Covid-19 pandemic, the interviews were conducted in a web-based format, lasting ∼40–80 min. Conducting interviews at least twice allows for a full understanding of scale-up-related complexities and gives researchers the opportunity to revise their findings at a later time [40]. For the present study, only data related specifically to scale-up were analyzed. Results on scientific collaboration are reported elsewhere [39]. A third round of interviews (n = 5) was conducted online by two researchers (RS; LB) from August 6^th^ to October 11^th^, 2021, and lasted ∼75–90 min. These interviews included specific questions about the choices made to achieve scale-up aims [41] to retrospectively assess to what extent the projects succeeded in reaching their goals for scaling up.

### 2.4 Data analysis

All interviews were audio-recorded and transcribed verbatim. RS performed a deductive content analysis [42] using MAXQDA software. RS created a codebook with a prime example for each code and translated all codes from German to English. LB analyzed the data, reviewed the codes, and verified translation accuracy. Discrepancies were discussed between RS and LB until agreement was reached. In the next step, the grant proposal was analyzed to identify the essential elements of scale-up and strategic scale-up choices for each project according to the WHO-ExpandNet framework [6]. Finally, all analyzed data were integrated into one set of results, which was discussed within the entire research group.

### 2.5 Ethical considerations

The Ethics Committee of the Friedrich-Alexander University Erlangen-Nuremberg granted ethical approval for this follow-up study (approval number: 390_18 B). All interviewees gave informed consent for the interview, the audio recording, and the scientific use of their data. All transcripts were anonymized so that no inferences could be drawn about the interviewees or others mentioned in the interview.

## 3. Results

In the pilot phase, the four projects (A4M, health.eduPLUS, PArC-AVE, QueB) implemented the participatory CP approach to 22 sites [33]. In the scale-up phase, the projects transferred it to 26 further sites, but the initial CP approach, as proposed by Rütten [15], was slightly adjusted within each project to meet the different agendas of its participants and available capacities of implementation settings [33]. Furthermore, each project developed and used its own scale-up strategy.

Based on the analysis, we identified key elements relevant for the scale-up process, which we categorized within the main two categories of the WHO-ExpandNet framework (i.e., innovation and user organization). Table 2 reports on the health innovations scaled up by the participatory projects and provides information on the implementers responsible for the process. The scale-up process in each project was collaboratively designed and planned by local representatives from the new implementation sites, such as administration officials in A4M and staff from nursing schools in PArC-AVE (refer to the “User Organization” row in Table 2) together with the researchers of the respective project team. These individual collaborations ensured that the scale-up plans were developed through ongoing interactions among researchers, policymakers, professionals, and population groups within the respective project team and implementation setting.

**Table 2:** Overview of innovations scaled up by the scientific projects, entities adopting the innovation and factors influencing scale-up across the projects.

WHO-ExpendNet elements *Definition*	A4M	health.eduPLUS	PArC-AVE		QueB
Attribute	Community	School	Nursing school	Car manufacturer	Childcare center
**Innovation**					
*The intervention and/or practices that were scaled up.*	The CP approach	The CP approach	The CP approach	The CP approach	A mobile app tool based on a certification procedure
*Other component(s) of pilot projects added to the scaled innovation*	*SportCard (i.e., a low-budget PA program* [25–27])	*Good practice examples for sport-related health competencies promotion*	*PA-related health competence learning content*	**–**	*Practice oriented learning tool (i.e., Quick-Assessment-Tool)* *Certification procedure*
**User Organization**					
*The institution adopting and implementing the innovation at scale.*	Local administration office of a community (*n* = 1)	Primary schools (*n* = 2)	Nursing schools (*n* = 2)	Car manufacturer (*n* = 1)	Childcare centers (*n* = 18)
Perceived need	(f) Local awareness among administration functionaries (f) Perceived need of functionaries to address PA in men 50+ (f) Community readiness for networking	(f) Motivated school staff acted as advocators of the innovation (f) Stakeholder readiness	(f) Health-oriented mindset of staff and students (f) Perceived need to address PA in vocational education	(b) Less perceived need for PA in employees	(b) Less organizational readiness in several centers (b) Less collective efficacy in several centers (f) Supportive staff in several centers
Implementation capacity	(f) Provision of personal resources (i.e., motivated functionaries) (f) Provision of financial resources (f) Provision of technical tools for dissemination (i.e., municipal internet portal) (f) Good coordination within the local administration office	(f) Political support from governmental representatives (f) Technical support (i.e., a ministry-run regional database as dissemination tool) (f/b) Different capacity for advertising the innovation in schools (b) Strict content requirements for introducing teaching concepts (b) Turnover of school staff	(f) Staff’s leadership (f) Political support	(b) Hierarchical decision-making processes (b) Drop-out of champion	(f) Implementation capacity provided by childcare funding organizations (f) PA-friendly structures of centers within rural areas (b) Less PA-friendly structures of centers within urban areas (b) Slow decision-making processes in several organizations (b) Less readiness for long-term processes (b) Less readiness for networking between childcare centers (b) Hierarchical decision-making processes (b) Less readiness towards long-term processes and outcomes
External collaborations	(f) Supportive local sports clubs (f) Supportive local adult education center (f) Supportive umbrella association for sports clubs (b) Local non-sports associations with less interest to promote PA	(f) Supportive political representatives of the Bavarian education authorities	(f) Supportive representatives of the Bavarian State Ministry of Education (f) Restructuration of the nursing training curricula		
External conditions	(f) New opportunities to promote PA among local sports clubs and adult education center (b) The Covid pandemic slowed implementation of the low-budget PA program	(b) The Covid pandemic slowed down PA promotion in schools (b) Content requirements for PA materials to be included on platform		(b) Work schedules of employees limited development of programs	

*CP = Cooperative planning; PA = Physical activity; (b) barrier; (f) facilitator; (f/b) both facilitator and barrier*.

Additionally, we identified various factors influencing the process, which we refer to as facilitators and barriers, assessing which of them influenced the need for scale-up, as well as the implementation capacity of those scaling the innovation. The following sections summarize the case studies of the four projects and provide an in-depth description of the scale-up plan, how the process was implemented, which strategies were employed to reach the scale-up aim (see Table 3), and which factors leveraged and/or hindered the scaling processes.

**Table 3:** Strategic management of scale-up across projects.

ExpandNet elements, attributes		A4M	health.eduPLUS	PArC-AVE				QueB
**Scale-up strategy**								
Horizontal	*Innovation’s component scaled up*	CP (12)	CP (12)	CP (12)	CP (12)			CP (12)
Vertical	*Innovation’s component scaled up*	Low-budget PA program [25–27]	Teaching content	Training curricula change				Training curricula change^*^
**Dissemination, advocacy**								
Collaboration(s)	*Local level*	✔	✔					
	*District level*	✔	✔	✔				✔
	*National level*							✔
**Organizational process**								
Academic-lead process		✔	✔	✔		✔		✔
Gradual, adaptive process		✔	✔	✔		✔		✔
**Resource mobilization**								
BMBF funding	*Regular (2018–2021)*	✔	✔	✔	✔		✔	
	*Extension (2021–2022)*	✔	(✔)	✔	✔		✔	
Others		Local financial support						
**Monitoring**								
Scale-up evaluation strategies		N/D	N/D	N/D	N/D			N/D
Others internal assessment types		✔	✔	✔	✔			✔

* the tool is at the experimental phase, being temporarily anchored as a practice-oriented learning tool in the training curricula of two nursery schools.

*BMBF stands (in German) for “Bundesministerium für Bildung und Forschung”/ The Federal Ministry of Education and Research*.

*N/D not defined.*

### 3.1 Case study 1: The “Action for men (A4M)” project

In the pilot phase, A4M used the participatory CP approach to improve PA in men over 50 in two rural communities. In the scale-up phase, the project aimed to transfer the CP process to a third community and engage local sports and non-sports associations (e.g., fire department) to jointly develop low-barrier PA offers for inactive men. The project collaborated with district and federal bodies (i.e., Health Administration of East Bavaria, Bavarian Medical Chamber) to disseminate new actions for PA promotion to other communities and regions.

#### 3.1.1 The scale-up process.

As CP was transferred to the new community, the local administration office was highly committed to introducing actions for PA promotion and providing sufficient implementation capacity (e.g., personal resources, such as motivated functionaries; financial resources; local network; technical tools for dissemination, such as a municipal internet portal). The local adult education center and the local sports clubs also pledged to implement additional low-threshold PA courses for inactive men in their daily sports programs. During the CP process, the local administration office was specifically interested in the PA promotion actions that had been successfully implemented in the pilot communities. As a consequence, the SportCard, a low-budget PA program [25,26] that had been developed within the CP process of one of the pilot communities, was directly introduced as part of the action plan for PA promotion in the new, third community. Unfortunately, its implementation was postponed due to the Covid-19 pandemic health regulations (e.g., social distancing). As an alternative, the community handed out an implementation guide to implement the SportCard [27] as soon as the pandemic allowed.

#### 3.1.2 Conclusion.

A4M adopted a combination of different scale-up strategies. As initially planned by the project, it horizontally scaled the CP approach, but also followed demands by the local administration to directly transfer another component of their pilot project (i.e., the SportCard). Additionally, A4M received political support from an umbrella association for sports clubs and used this collaboration to design regional workshops for PA and sport trainers on encouraging low-threshold PA among men over 50. The project succeeded in vertically “anchoring” this concept within the training structures of collaborating institution on the district level, proposing the SportCard as a potential solution for wider regional PA promotion.

### 3.2 Case study 2: The “health.eduPLUS” project

In the pilot phase, the project used the CP method to develop actions improving the sport-related health competencies of secondary school children, as well as the teaching competencies of physical education (PE) students. The scale-up phase focused solely on the scale-up of CP to four primary schools. Based on the experience from the pilot phase, the goal was to develop good practice examples for PE teachers to promote sport-related health competencies. The project also sought support from the Bavarian education authorities to disseminate these examples to PE teachers across Bavaria using a governmental PE teaching platform.

#### 3.2.1 The scale-up process.

While transferring CP to the four new schools, school staff (e.g., principals, junior PE teachers in training, and advanced skills teachers) were motivated to different extents to make changes, with those familiar with the pilot project acting as driving forces. Teacher participation in CP meetings also varied across the four schools, mostly based on how the principals internally advertised the project. Overall, the turnover of school personnel and the priority of regular school activities over optional projects impacted the implementation of CP in the schools. Due to the new health regulations created by the Covid-19 pandemic (e.g., home schooling), there was less focus on promoting PA among the schools, which in turn influenced the scale-up process. Several government representatives participated in CP meetings and offered support, such as making the ministry-run regional teaching database available to include training examples for sport-related health competencies. However, even though the content was jointly developed by scientists, school staff, and political representatives of the Bavarian education authorities, it eventually failed to meet all of the platform’s formal content requirements and was thus unable to be included.

#### 3.2.2 Conclusion.

The project adopted a mix of scale-up strategies. It horizontally scaled up CP to another school type (primary school) compared to those in the pilot phase. It additionally planned vertical strategies to institutionalize specific components of their pilot project as educational norms and guidelines. The goal of the latter strategy was to achieve change at higher educational levels by making sport-related health competence a teaching component accessible to all Bavarian PE teachers on a lasting basis.

### 3.3 Case study 3: The “PArC-AVE” project

In the first funding phase, PArC-AVE used CP processes to address PA promotion in the vocational setting, namely in a car manufacturing company and a private nursing school. In the second phase, the project was extended to the production division of the car manufacturing company and two state-run nursing schools. One aim was to transfer the CP and use it to develop strategies to integrate PA promotion into regular work activities of the new organizations; the other was to include PA-related health competence into the curriculum of state nursing schools with support from Bavarian State Ministry of Education.

#### 3.3.1 The scale-up process.

Prior to scale-up, the PArC-AVE scientific team applied the “practice-dive approach” [29] to temporarily immerse researchers in the setting and familiarize themselves with the environment and population. While not explicitly planned to be part of the scale-up process, this approach unintentionally enhanced researchers’ credibility in the eyes of the target population, thus improving the scale-up process.

As CP was transferred to two state-run nursing schools, the schools already showed a strong health-oriented mindset. Participating actors such as staff, professionals, and trainees saw a need to promote PA, and trainers displayed empowering leadership. Other participants, such as representatives of the Bavarian State Ministry of Education, were supportive to sustainably anchor the topic of PA-related health competencies in the curriculum.

In the car manufacturing setting, the transfer of CP from the training to the production division was intended to target employees who would benefit most from PA. An influential “champion” in the company initially drove the process but left during the scale-up phase, leaving no one with a similar commitment to take over. Employees from the production division saw less need for PA than nursing school staff, probably as their priorities were naturally on production output rather than health. Regarding the CP process, tight production schedules made it hard to exempt workers from their duties to participate in the development of PA promotion actions. Compared to the nursing schools, it seemed generally more difficult to find ways to implement PA promotion actions given the more hierarchical communication and decision-making processes of a for-profit company.

#### 3.3.2 Conclusion.

The project succeeded in the horizontal scale-up of the CP approach to three new sites. For the nursing school settings, there were also favorable circumstances for a vertical scale-up. This was achieved by institutionalizing a specific pilot-phase component, i.e., the “PA-related health competence” topic, into the vocational education curriculum for nurses in Bavaria. Political support from the Bavarian Ministry of Education, underpinned by the perceived need for an urgent restructuring of the nursing training curriculum, offered an opportunity for change in the educational program to permanently address PA-related health competencies in state-run nursing schools.

### 3.4 Case study 4: The “QueB” project

In the pilot phase, QueB intervened in twelve childcare centers located in two regions, where they applied the CP approach, followed by counseling and/or evaluation sessions on how those centers can promote PA among their staff and children. During the scale-up phase, QueB reached out to other childcare centers located in difficult social environments, characterized by challenging social circumstances, such as poverty and/or a large population of children with a migrant background. QueB aimed to scale-up the CP, but also actions developed within the pilot phase: a mobile app (the Check-App) and a certification procedure to annually assist centers on how PA-friendly they are. To achieve a wide roll-out, QueB established a close collaboration with a national platform for diet and PA promotion to create a network of stakeholders at the regional level. The project focused on training instructors by empowering them to facilitate PA promotion in other childcare centers in the region, especially those with low PA levels and hard to reach.

#### 3.4.1 The scale-up process.

In the scale-up phase, the CP approach was used to introduce the mobile app and slightly adapt it to the requirements of the staff and childcare center. It was followed by the attempt to scale the mobile app together with the certification procedure to 18 new childcare centers located in areas characterized by challenging social circumstances. Many staff members of centers within urban areas were not well-prepared to prioritize PA activities due to the competing priorities within the centers, such as addressing the language difficulties of children with migrant background. This situation resulted in lower organizational readiness for change, i.e., defined as collective beliefs, attitudes, and behaviors of members regarding their willingness to implement new initiatives or transformations [43] and a diminished sense of collective efficacy regarding PA promotion.. However, some centers (i.e., mostly those located in rural areas) were highly motivated to utilize the mobile app and ensured the capacity to implement it. Moreover, the attitude of the centers’ parent institutions towards PA was a key factor in ensuring resources and support for a successful scale-up. However, the complex hierarchical structure of these organizations often slowed down their decision-making process, leading to slower scale-up at times. Furthermore, some organizations appeared to lack the stamina for long-term processes and transformations. QueB also aimed to establish a network of childcare centers to create a mutual learning process. Interestingly, many centers displayed a low readiness to collaborate with each other, perceiving each other as competitors rather than potential collaborators.

#### 3.4.2 Conclusion.

The project partially succeeded in scaling up the mobile app and certification procedure to help childcare centers become PA-friendly. Within Region A, the mobile app was adapted to a practice-oriented learning tool (called the Quick Assessment Tool) that was horizontally scaled to seven childcare centers. Additionally, the support offered by the parent institutions of these centers enabled the vertical scale-up of the Quick Assessment Tool into the regional vocational education program for future nursery teachers. In Region B, the mobile app focused on teaching modules. Using a 10-step certification procedure, it was implemented in twelve childcare centers located in various rural and urban areas. Centers in rural areas had more favorable environments for outdoor activities (i.e., greater physical space for PA such as large gardens and green landscapes, as well as nearby forests) compared to those in urban regions, and could successfully uptake the teaching modules to improve their PA standards. However, centers in urban areas had poorer social conditions (e.g., poverty, social disadvantaged neighborhoods) and were less capable of implementing actions supporting PA promotion. Due to lack of funding, the scientific team that initially developed the mobile app could not provide them with closer support, which consequently caused five of them to drop out of the scale-up process early.

## 4. Discussion

Closing the gap between what we know about scale-up, what frameworks are recommended for the process, and how scale-up of participatory innovations actually takes place demands a deep understanding of the complexity of participatory projects and how they fit into different contexts. We investigated the different scale-up experiences of four participatory projects to gain insights into what scale-up looks like in the real world: The process can follow a strategic plan, but it requires scientific expertise for implementation, flexibility to continuously be tailored to local contexts, and a certain intuition to take advantage of “windows of opportunity” to integrate PA promotion into existing systems or structures.

### 4.1 Central findings and implications for scaling up participatory projects

First of all, our results yielded useful insights regarding the “innovation” of participatory projects and strategies on how to transfer (horizontal scale-up) and institutionalize (vertical scale-up) them. In our study, most projects referred to the participatory CP method as the innovation planned to be scaled, but results show that other components of their pilot projects were also scaled to new sites. Most projects scaled up the CP process *horizontally* to new sites (different schools, company division, communities, childcare centers), but at the same time, many researchers utilized other components of their pilot projects (i.e., PA promotion actions successfully developed in the pilot phase – see examples in Table 2) to (a) transfer them directly to the new sites, and (b) subsequently scale them *vertically* within organizational structures and systems.

For example, in the A4M project, the CP approach was transferred to a third community, but instead of developing new low-barrier PA offers from scratch, local stakeholders opted to transfer and re-implement specific PA actions (i.e., the SportCard) that had been successfully implemented in the pilot communities of the project’s pilot phase. This strategy emerged from a collaborative decision-making process within the A4M project, involving the researchers and the other participants who were eager to transfer the successful PA strategy from the pilot phase. They collectively acknowledged that this strategy aligned with the capacities and needs of the new community. This result underscores the significant influence of context factors – such as cultural beliefs, local politics and/or available resources – can have on the scale-up process.

Such previously tested PA promotion actions may be less disruptive and easier to implement, and can thus have a substantial and immediate positive impact on the target setting. However, such a strategy bypasses a core tenet of CP and no longer aligns with its original goals (see Section 2.1.) regarding empowerment and structural changes [15].

In terms of participatory research, one might argue that the process shifts from a bottom-up to a top-down approach when changing the perspective on the innovation to be scaled, i.e., when adopting tested PA promotion actions directly (innovation=actions for PA promotion; as described in the case of the A4M project) rather than involving population groups intensively in their planning and development (innovation=CP; as in the case of the PArC-AVE project). Also, it no longer aligns with the WHO’s recommendations on working multisectorally and transdisciplinarily in order to develop solutions that have the power to alter structures at a systemic level and are disruptive enough to trigger sustainable change processes [44]. On the other hand, it may be well-suited to the current era of complex problem situations and challenges (e.g., climate change, pandemics) that requires solutions leading to rapid change in population behavior. This desire for “simpler” actions that lead to quicker changes is apparent in the third community of A4M, which skipped much of the CP development phase to directly implement actions that provided people with faster access to PA. In addition, as participatory processes like the CP require substantial resource allocation to engage members of the population group, policymakers, and practitioners [33], the direct scale-up of specific actions may often be considered a much cheaper (and thus much more attractive) option by community leaders.

However, it must be noted that the experience from Capital4Health indicates that the CP should not be abandoned completely in favor of a direct scale-up of actions for PA promotion. Rather, the CP is merely given a slightly different role within the scale-up process: It is still used to facilitate the original participatory development of actions, and many projects will first repeat the process at new sites as intended by the original concept. However, as more and more successful actions become available, there may be a shift from repeating the process towards directly transferring actions to other, comparable sites, see for example the study of Herbert-Maul et al. [13]. This tradeoff may be considered acceptable as (a) the actions are rooted in a participatory process and (b) the transformation process is constantly gaining pace. One may conclude that it could be useful to build a gradual change of focus regarding the innovation (from the participatory process towards its actions) into the scale-up concept of future participatory projects right from the start.

Another important finding pertains to the relationship between horizontal and vertical scale-up in participatory projects. Available research emphasizes that horizontal scale-up alone is not sufficient to ensure that people benefit from the innovation on a long-term basis – vertical strategies are needed for “policy, political, legal, regulatory, budgetary, or other health systems changes” [6] to achieve sustainability. This was illustrated by health.eduPLUS and PArC-AVE, both of which transferred (i.e., scaled horizontally) the CP method to new sites but used specific actions (e.g., PE content lesson, PA promotion concepts) to try to institutionalize (i.e., scale vertically) these actions to the state teaching curriculum and good practice database, respectively. Experience from other projects also suggests that specific PA actions developed within participatory processes can only be successfully integrated into local health promotion structures if they are developed in a participatory manner [13]. However, as in the case of a direct transfer of actions to other sites, health promoters need to be aware that the increased reach of such institutionalized actions comes at the price of reduced “tailoring” to specific populations.

### 4.2 Strengths and limitations of the study

Several limitations to this study need to be mentioned: Generalizability of our results is limited, especially as some of our settings are unique to the German system (i.e., vocational training in companies, school types). Nonetheless, the similarity of our results to other studies [1,45] points towards essential aspects to consider in future strategic planning processes for enhancing scalability, regardless of country background [6,46,47].

Another shortcoming of our study relates to the quality criteria of reporting qualitative results [36]. During the interviews, some projects identified more key factors than others. By not returning our findings to the interviewees, we missed a chance for further improving our data. A possible solution for future research would be to send interviewees the questions in advance to enhance reflection of past events and help them better prepare for the interviews.

There are also some limitations related to our use of the WHO-ExpandNet framework. The ExpandNet was originally not developed for retrospective analysis, as done in this and other studies, e.g., [37], but as a prospective guideline for scaling interventions and increasing project impact. This presented several challenges for our analysis, particularly in terms of handling the complexity of the framework while considering the two purposes mentioned above. In our research, we had to streamline a part of the framework, focusing only on the key elements that were relevant for making affirmations related to the scaled innovation, the organizations and individuals responsible for implementation, their environments and scale-up strategies. Despite this decision, our results indicate that we were still able to provide a detailed description of the scale-up processes, mostly regarding the various ways of scaling participatory projects. However, it is worth noting that the ExpandNet framework has limitations in capturing the diversity of these strategies, necessitating conceptual adaptations when using the framework for participatory research.

## 5. Conclusions

Scaling up is a complex process, mostly because projects are expected to meet several goals all at the same time, i.e., following a strategic plan, continuously adjusting to emerging situations within new sites, and taking advantage of given opportunities (e.g., political, structural) to sustainably anchor PA promotion at the system level. Our study shows that participatory projects for PA promotion use different innovative approaches in order to *horizontally* and *vertically* scale multiple project components to new sites. Based on their experience, one can deduce several recommendations for health promoters who wish to scale up participatory projects:

First, it is essential to be clear about the *innovation(s)* to be scaled up: This could be the participatory method (such as the CP), specific actions for PA promotion emanating from the use of this method in the pilot phase, or a combination of both. The main focus of scale-up may vary between projects or even change over time within a specific project, but having a clear plan from the outset may facilitate the process.

Second, the decision on a particular set of innovations should be guided by several considerations, particularly concerning the available resources and the appropriateness of reducing the participatory element: A direct transfer of existing actions is likely to be much cheaper than a transfer of the participatory method, and thus has a much higher likelihood of achieving a truly widespread scale-up. By contrast, one has to make sure that these ready-made actions, even if they were originally co-developed, are still appropriate for the new setting and population group. Critical components of participatory projects need to be identified, and the whole scale-up process has to be monitored and assessed in terms of reach and impact. Otherwise, projects run the risk of losing the benefits associated with a participatory approach or even inadvertently worsening health inequalities as highlighted in the literature on the inequality paradox [48].

Finally, there may be a need to critically revise available frameworks for scale-up with respect to their suitability for participatory projects. While ExpandNet proved to be a good starting point for our analysis, it may need to be amended or replaced by novel theoretical concepts of scalability that better capture the diversity of participatory scale-up approaches observed in our study.

The findings of this study suggest several practical implications for future research on scaling participatory innovations for PA promotion. For example, more flexible scale-up methods are needed to adapt participatory PA innovations to the unique characteristics and needs of various sites, such as difference between urban and rural settings or varying school or institution structures. Developing adaptable frameworks that account for diverse local contexts can improve both scalability and long-term sustainability. Since settings vary in their capacities, resources and constraints, future research should prioritize tracking and assessing how interventions/innovations are customized during the scale-up phase. This may involve creating tools to measure the impact of context-specific adaptations on outcomes, ultimately helping to refine guidelines for effective scaling.

Additionally, it is essential to develop new theoretical concepts of scalability that reflect the diverse characteristics identified within the participatory projects examined in our study. These findings can inform future research on strategies to enhance the effective implementation and scaling of participatory physical activity innovations, ensuring they remain adaptable and sustainable across different settings.
